# Development and identification of fully human scFv-Fcs against *Staphylococcus aureus*

**DOI:** 10.1186/s12865-016-0146-z

**Published:** 2016-04-29

**Authors:** Siji Nian, Tong Wu, Yingchun Ye, Xu Wang, Wenfeng Xu, Qing Yuan

**Affiliations:** The School of Basic Medical Sciences, Sichuan medical university, Room 218, Hanguang building, No 319, Zhongshan road, Luzhou, Sichuan 646000 China

**Keywords:** *Staphylococcus aureus*, Single-chain variable fragment, Fragment crystallizable regions, scFv-Fc, Phage display

## Abstract

**Background:**

*Staphylococcus aureus*, a gram-positive pathogen, causes many human infections. Methicillin-resistant *S. aureus* (MRSA) is the most common drug-resistance bacteria. Nearly all MRSA bacteria are resistant to several drugs. Specific antibodies are the main components of the host’s humoral immunity, and play a significant role in the process of the host’s resistance to bacterial infection.

**Results:**

A single-chain variable fragment (scFv) library was constructed using mRNA from the peripheral blood mononuclear cells of *S. aureus* infected volunteers. After the scFv library DNA was transformed into *Escherichia coli* TG1, ~1.7 × 10^7^ independent clones with full-length scFv inserts. The scFv library was screened by phage display for three rounds using *S. aureus* as an antigen. The single clones were chosen at random and the scFvs were expressed for enzyme-linked immunosorbent assay (ELISA) assessment. Approximately 50 % of the clones were positive with good binding activity to *S. aureus*. To improve the stability of scFvs, scFv-fragment crystallizable regions (-Fcs) were constructed and expressed in *E. coli* DH5α. The expressed scFv-Fcs were purified and identified by western blot. These antibodies were further characterized and analyzed for bioactivity. The results showed that the expression level and folding of scFv-Fcs induced at 25 °C without isopropyl β-D-1-thiogalactopyranoside (IPTG) were higher than that induced at 32 °C with 1.0 mmol/L IPTG. scFv-Fcs had good bioactivity and could specifically bind with *S. aureus*.

**Conclusion:**

scFv-Fcs against *S. aureus* were successfully constructed and are good candidates for the development of future adjunctive therapy for severe *S. aureus* infections.

## Background

*Staphylococcus aureus*, a gram-positive pathogen, causes an large array of diverse human infections, ranging from relatively minor skin and wound infections to more serious life-threatening diseases, such as deep-tissue infections, pneumonia, and bacteremia [1, 2]. Based on data from the U.S Centers for Disease Control and Prevention, *S. aureus* infection has been deemed the most lethal of all infectious diseases. The emergence and spread of multidrug-resistant strains in communities and even in hospitals are making therapeutic intervention increasingly difficult and expensive [3–5]. Meticillin-resistant *S. aureus* (MRSA) is the most common drug-resistant bacteria and causes clinical diseases such as skin infections, pneumonia, and septicemia. Nearly all MRSAs are also multidrug resistant and are not sensitive to β-lactam antibiotics, such as penicillin and cephalosporins, or to chloromycetin, lincomycin, aminoglycoside antibiotics, tetracycline antibiotics, and macrolide antibiotics. Some *S. aureus* are also not sensitive to vancomycin [6–9]. With only a few new antibiotics in development, considerable interest and efforts have been directed toward exploring active and passive immune-mediated therapeutic approaches to prevent and treat staphylococcal infections.

Specific antibodies are the main component of a host’s humoral immunity, and play a significant role in the process of creating a host’s resistance to bacterial infection. Antibodies can bind with the antigens of bacteria and kill or eliminate the bacteria through neutralization, activating complements, promoting phagocytosis, and antibody-dependent cellular cytotoxicity (ADCC). For nearly 30 years, antibodies have gone through four stages of development—murine antibodies, human-mouse chimeric antibodies, humanized antibodies, and fully human antibodies. Fully human antibodies are used to reduce the rejection reaction when treating human diseases [10, 11].

Traditional monoclonal antibodies were murine. If used to treat human diseases, the human anti-mouse antibody (HAMA) reaction would occur; therefore, fully human antibodies were perfected to treat human diseases. A fully human antibody is prepared in one of two ways—using transgenic mice or constructing a human phage single-chain variable fragment (scFv) library. The molecular weight of the fully human antibody is too high to use phage display for selection. scFv has the property of low molecular weight, which is suitable for phage display, and has antigen-binding properties; therefore, scFvs is the best block for constructing other types of antibodies. However, scFvs are not stable and do not have the properties of fragment crystallizable regions (Fcs), such as ADCC, so Fcs were fused with scFvs, creating scFv-Fc, to increase stability and recover Fc function.

In this study, an scFv library was constructed using mRNA from peripheral blood mononuclear cells (PBMCs) of volunteers infected with *S. aureus* and specific fully human scFv-Fvs against *S. aureus* were developed with the hope that an adjunctive therapy is developed for severe *S. aureus* infections.

## Methods

PBMCs of the five volunteers enrolled in this study who were infected with *S. aureus* were used for the construction of a scFv library. The criterion for selecting volunteers was only that they were infected with *S. aureus*, which was confirmed by tests from the clinical laboratory in the first affiliated hospital of Sichuan medical university, China. The experiment was approved by the ethics committee of Sichuan medical university (No. 5105025012142). All volunteers were adults and provided written informed consent.

### Construction of the scFv library

The scFv library was constructed by referring to previous reports by our research group [12]. Briefly, PBMCs from volunteers infected with *S. aureus* were isolated by Ficoll Paque Plus (Amersham Pharmacia Biotech, Inc., Piscataway, NJ, USA) according to the manufacturer’s instructions. mRNA was extracted from the PBMCs using the Dynabeads mRNA DIRECT kit (Invitrogen, USA) and was used to synthesize full-length cDNA using the SMART cDNA library construction kit (Clontech, USA). The variable regions of heavy-chain (V_H_) and light-chain (V_L_) immunoglobulin (Ig) were amplified by four subsequent polymerase chain reactions (PCRs) using a set of primers and following Qing Yuan [12] and the V_H_ and V_L_ gene repertoires were linked by overlapping extension PCR [12].

The phage display vector pCANTAB-5E, *Escherichia coli* strain TG1, and helper phage M13K07 (Amersham Pharmacia Biotech, Inc., Piscataway, NJ, USA) were used to create the phage library. The scFv library DNA (V_H_-linker-V_L_) and phagemid pCANTAB-5E vector were digested with *Sfi* I and *Not* I. The digested scFv fragments were inserted into the vector to generate a scFv-gene III fusion library using T4 DNA ligase (New England BioLabs, UK) at 4.0 °C overnight. The ligated products were transformed into *E. coli* TG1 by electroporation and grown at 37 °C on culture plates containing lysogeny broth (LB) medium supplemented with 100 μg/mL ampicillin and 2.0 % glucose. Thirty clones from the library were selected at random and identified by PCR to estimate the proportion of full-length scFv clones. The PCR products were digested by *BstN* I for producing a fingerprint map to estimate the diversity of scFvs. All clones on the plates were scraped and suspended in LB containing 15 % glycerol.

### Phage amplification

Eighty microliters of scraped bacterial cells or frozen cell suspensions under glycerol were incubated in 40 mL LB containing 100 μg/mL ampicillin and 2.0 % glucose until the optical density at a wavelength of 600 nm (OD_600_) = 0.2 while shaking at 37 °C. The bacteria were collected by centrifugation and suspended in 40 mL LB with ampicillin without glucose. Approximately 6 × 10^9^ transducing unit (TU) of helper M13K07 (Amersham Pharmacia Biotech, Inc., Piscataway, NJ, USA) were added to each milliliter of cell suspension, incubated for 15 min at 37 °C without agitation, and incubated for another 2.0 h with agitation. Kanamycin was added to obtain a final concentration of 20 μg/mL, after which the cells were incubated overnight at 32 °C. The phage was subsequently precipitated with polyethylene glycol (PEG)-NaCl (20 % PEG, 2.5 mol/L NaCl) and resuspended in 0.01 mol/L phosphate-buffered saline (PBS) buffer.

### Affinity selection

Immunotubes were coated with ~1.0 × 10^8^ cfu/mL *S. aureus* in coating buffer (0.1 mol/L Na_2_CO_3_/NaHCO_3_, pH 9.6) and left overnight at 4.0 °C; these were then sequentially blocked with 4.0 % fat-free milk for 1.0 h at 37 °C and gentle agitation. Approximately 1 × 10^12^ TU from the freshly amplified scFv libraries were added to the blocked immunotubes and incubated at room temperature for 2.0 h. The immunotubes were rinsed 10 times with 0.01 mol/L PBST (0.05 % Tween-20 in 0.01 mol/L PBS) and 0.01 mol/L PBS separately, then eluted with 0.1 mol/L glycine-HCl (pH 2.2) and amplified as above. In the second and third rounds of affinity selection, 1 × 10^7^ cfu/mL and 1 × 10^6^ cfu/mL bacteria antigen, respectively, were coated and the immunotubes were washed separately 15 to 20 times with 0.01 mol/L PBST and 0.01 mol/L PBS.

### Phage enzyme-linked immunosorbent assay

Following affinity selection of the scFv library in three rounds, individual clones were randomly chosen and grown at 37 °C. The phage was amplified according to the previously described protocols. The amplified phage preparation (10^12^ TU) was blocked with 4.0 % fat-free milk in 0.01 mol/L PBS for 30 min and added to an immunoassay plate coated with *S. aureus* for enzyme-linked immunosorbent assay (ELISA) and the negative control (0.01 mol/L PBS) was set up. Plates were incubated for 1.0 h, washed three times with washing buffer PBST, and finally incubated with horseradish peroxidase (HRP)-conjugated anti-M13 mouse monoclonal antibody (Amersham Pharmacia Biotech, Inc., Piscataway, NJ, USA). The immunoreactions were developed by incubating in TMB liquid substrate (Sigma-Aldrich Company, Inc., St. Louis, MO, USA) for 15 min. The reactions were stopped by adding 2.0 mol/L H_2_SO_4_. Absorbance at 450 nm was recorded and used to select the phage that expressed scFv for recognizing the target antigens.

### Construction and expression of scFv-Fcs

**Fig. 1:**
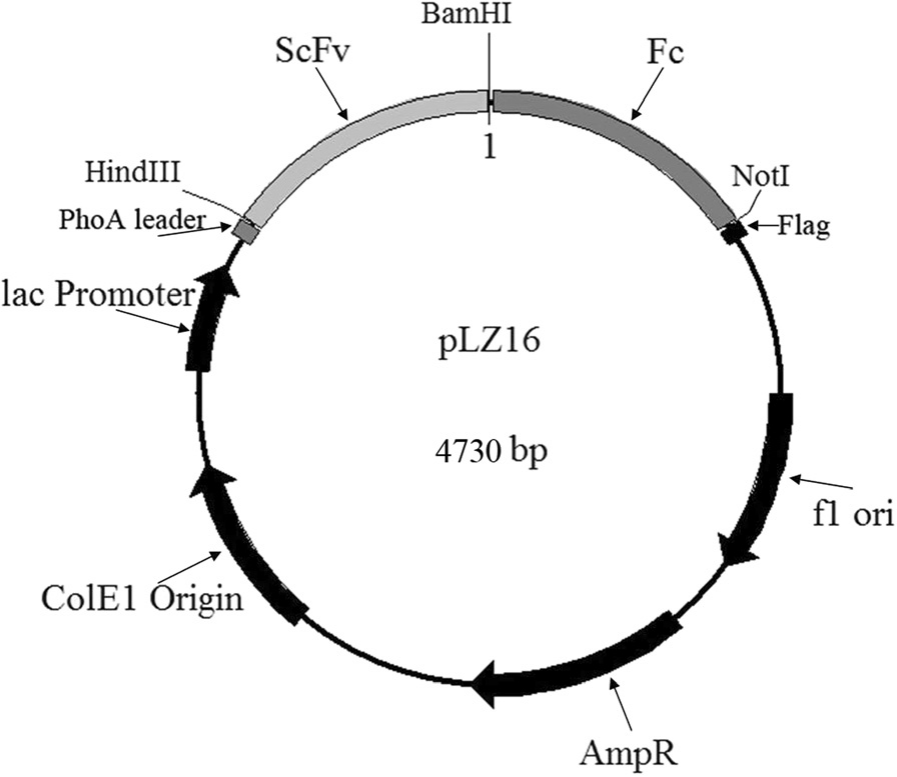
Plasmid map of pLZ16. The vector was constructed based on the pUC vector and used for soluble single-chain variable fragment- fragment crystallizable region (scFv-Fc) production

pLZ16 was designed based on pUC vector for soluble scFv-Fc antibody production by introducing leader peptide-encoding sequencing (PhoA leader), cloning sites (*HindIII*, *BamHI*, *NotI*) and FLAG tag. The Fc was inserted into the plasmid (Fig. 1). The selected scFvs with good binding activity and specificity were ligated with the pLZ16 vector and transformed into DH5α cells of *E. coli*. The positive clones were cultured to OD_600_ = 0.5 at 37 °C in LB with 100 μg/mL ampicillin, 2.0 % glucose (LBAG) and then centrifuged at 5000 r/min for 10 min. One of the following two techniques was used to induce scFv-Fcs expression. One technique was to resuspend the pellets with LBA containing 2.0 mmol/L MgCl_2_ and culture at 25 °C for 50 h [13]. The other was to resuspend the pellets with LBA and 1.0 mmol/L IPTG and culture at 32 °C for 5.0 h. After centrifugation, the pellet was collected and resuspended in 0.01 mol/L PBS. The suspended cells were sonicated and centrifuged, and the supernatant was detected by ELISA and western blot.

### scFv-Fc functionality

The binding activity of and specificity of scFv-Fcs to *S. aureus* was tested by dot blot and ELISA using the anti-human IgG1 Fc antibody. Briefly, *S. aureus, S. albus, S. citreus, Salmonella typhi, Bacillus anthracis,, Shigella Castellani, Proteusbacillus vulgaris, Monilia albican, α-hemolytic streptococcus* and *β-hemolytic streptococcus* was suspended in Na_2_CO_3_/NaHCO_3_ coating buffer at a concentration of 10^7^ cfu/mL and the ELSIA plates were coated at 4.0 °C overnight or the nitrocellulose membrane was dotted for dot blot analysis. The scFv-Fcs were purified and 1 μg/mL purified scFv-Fcs were used as first antibody and the anti-human IgG1 Fc antibody was used as second antibody for test. The dot blot immunoreactions was developed by chemiluminscence and the ELISA immunoreactions was developed by TMB substrate.

The binding activity of scFv-Fcs were also analyzed by BIAcore (BLITZ). The scFv-Fcs were immobilized on the anti-hIgG Fc capture (AHC) biosensor. Then the *S. aureus* with different concentration (1 × 10^7^ cfu/mL, 2 × 10^7^ cfu/mL, 4 × 10^7^ cfu/mL) were added and the association and dissociation of scFv-Fcs and S. *aureus* were detected. The 0.01 mol/L PBS were used as control.

The opsonization of scFv-Fcs was researched by testing the phagocytosis of antibody-opsonized pathogens. The peritoneal macrophages of mice were isolated. The macrophages and *S. aureus* (1 × 10^7^ cfu) was mixed at the ratio 1:20 in RPMI-1640 medium containing 10 % fetal calf serum (FCS). Ten microliter of scFv-Fc (100 μg/mL) was added into the mixture and incubated at 37 °C for 2 h with gentle agitation. The mouse IgG1 was used as control and each group was repeat for three times. Then the 200 μL of mixture was spread the LB plates for overnight culture. The following day, the clone numbers were calculated.

### Statistical method

Background noise correction was performed from ELISA by subtracting the absorbance. All the data were repeated for three times and the data sets the mean and the standard error were calculated. Data were presented as means ± SEM. Differences between groups were determined by the two-tailed *t*-test. P < 0.05 was considered statistically significant. *p < 0.05, **P < 0.01, ***P < 0.001.

## Results and discussion

### Construction of the scFv library

**Fig. 2:**
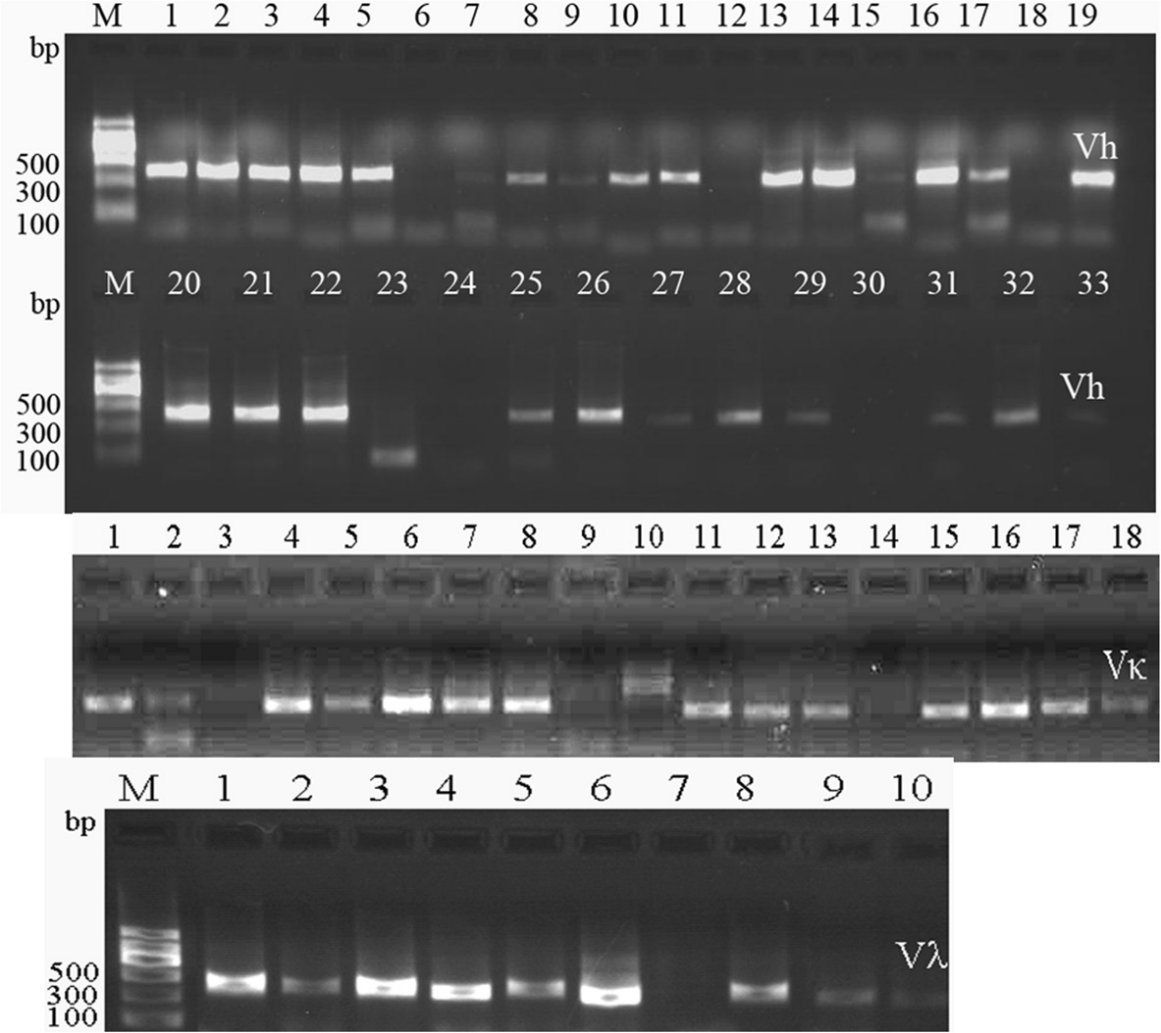
Amplification of V_H_, V_κ,_ and V_λ_ gene repertoires. Lane M: DNA Marker II (Tiangen, CN). The expected size of the amplified V_H_ and V_L_ (including V_κ_ and V_λ_) was ~350 bp. A part of amplified V_H_, V_κ_, and V_λ_ were shown in Fig. 2

mRNA was extracted from PBMCs of volunteers infected *S. aureus*. The quality of the mRNA was the key to synthesize the full-length cDNA. The integrity of the extracted mRNA was determined by testing the OD_260_/OD_280_ and electrophoresis. The synthesized ds cDNA appeared as a smear of from 0.1 to 8.0 kb on the gel and the size of distribution, yield, and intensity of cDNA were satisfied (data not shown). The full-length cDNA was used as a template to amplify the regions of V_H_ and V_L_, including V_κ_ and V_λ_. The V_H,_ V_κ_, and V_λ_ gene repertoires were amplified with 42, 16, and 18 optimized primer sets, respectively. Nearly all PCR reactions were effective and the correct PCR products were obtained from the gel picture; the expected size of the amplified V_H_ and V_L_ (including V_κ_ and V_λ_) was ~350 bp. A part of amplified V_H_, V_κ_, and V_λ_ were shown in Fig. 2. The V_H_-linker gene and V_L_-linker gene repertories were amplified and the V_H_-linker-V_L_ gene repertories (750 bp) were obtained by overlapping extension PCR. The DNA library of human scFvs was developed.

**Fig. 3:**
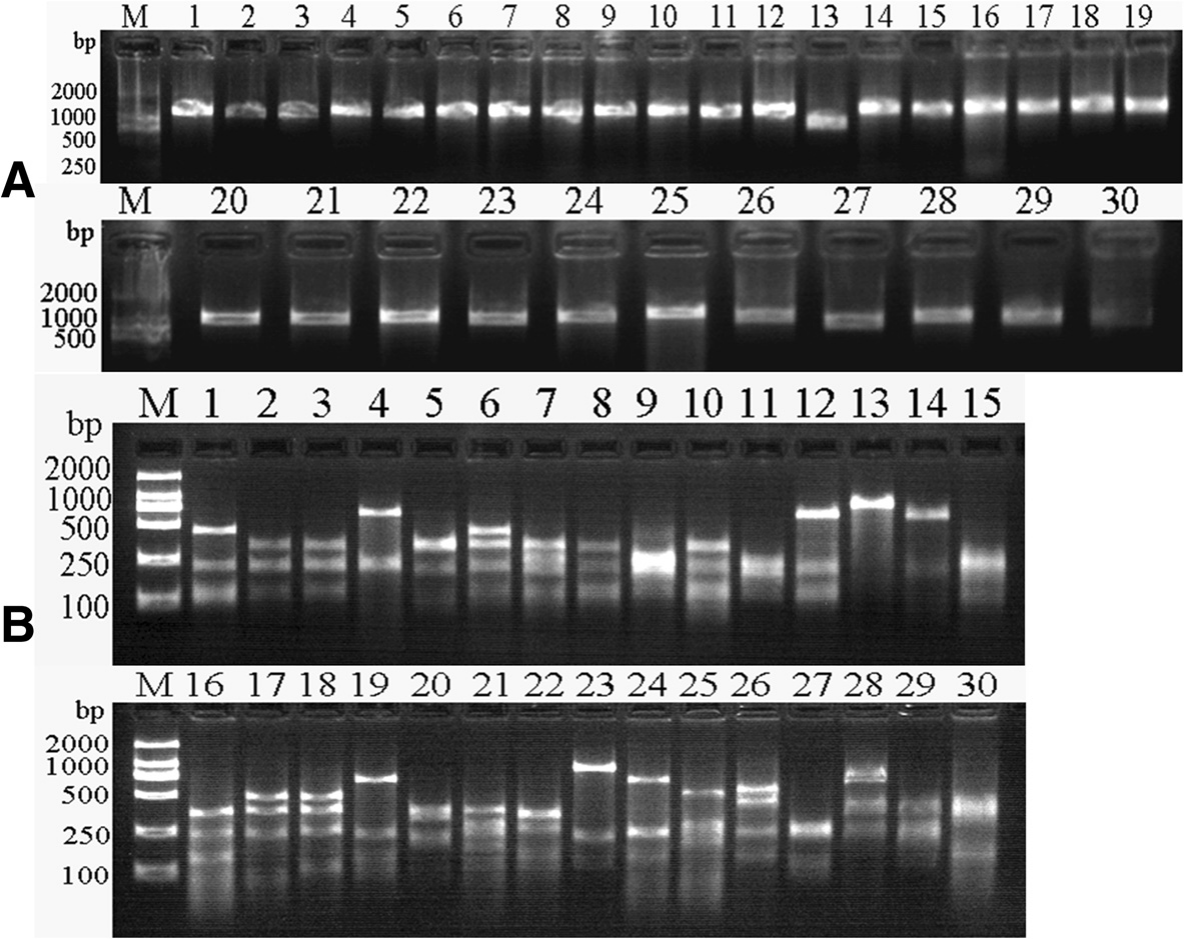
**a** Thirty clones were chosen randomly from the single-chain variable fragment (scFv) library and the positive clones inserted into full scFv genes were identified by polymerase chain reaction (PCR). Lane M: 2000 bp DNA Marker (TaKaRa, JP). Lanes 1–30: amplified scFvs from different clones randomly picked. **b** PCR products of scFvs were digested by *BstN* I. 2000 bp DNA Marker (TaKaRa, JP). Lane M: 2000 bp DNA Marker (TaKaRa, JP). Lanes 1–30: Fingerprints of scFvs digested by *BstN* I

**Fig. 4:**
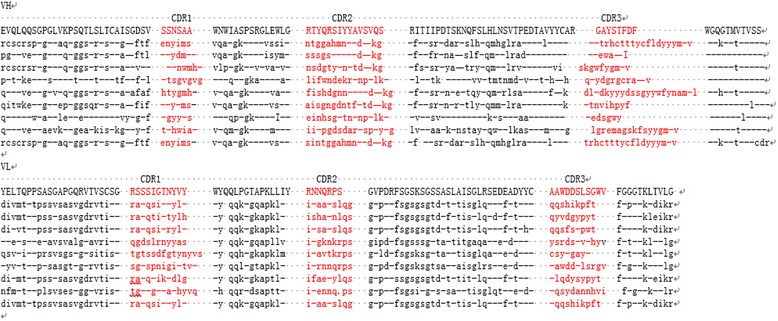
Amino acid sequences of 10 clones which were randomly picked from the library and analyzed by DNA sequence

The DNA of the scFv library was ligated with phagemid vector pCANTAB-5E and transformed into TG1 cells of *E. coli*. Thirty clones were randomly chosen and the inserted scFvs were identified by PCR. The results showed that all clones contained full-length scFv genes. By *BstN* I digestion of the scFv DNA, the fingerprint maps showed that almost all scFvs were different (Fig. 3); For further analysis of the diversity of the library, the 10 clones were picked randomly for sequencing. The results showed that the sequences of those scFvs were diverse (Fig. 4). The library was estimated to contain 1.7 × 10^7^ unique scFv members.

### Affinity selection

The phage library was amplified and for each round of affinity selection, ~10^12^ TU freshly amplified scFv antibody library were used for screening. Approximately 1.1 × 10^6^, 3.1 × 10^6^, and 1.2 × 10^7^ phages were recovered after the first, second, and third cycles, respectively. Phage pools before and after each round of affinity selection were tested by ELISA, which was performed by using the anti-M13 HRP-conjugated secondary antibody. The phage pool before affinity selection had no signal with the antigen, but after three rounds of screening, there was strong binding activity between the library phage and antigen (data not shown). Hundreds of clones were randomly chosen from the phage pool after three rounds of affinity selection. The phages amplified from those clones were tested by ELISA and about 50 % showed positive whose absorbance of OD_450_ was two-fold those of the negative controls.

### scFv specificity test

**Fig. 5:**
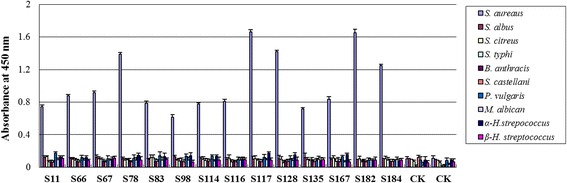
The specificity of selected single-chain variable fragment (scFvs) detected by enzyme-linked immunosorbent assay*.* Number 15 and 16 were negative controls

From the hundreds of clones those with higher absorbance had better binding activity to the antigen and were selected for testing scFv specificity. The *S. aureus, S. albus, S. citreus, S. typhi, B. anthracis*, *S. castellani, P. vulgaris, M. albicans, α-H.streptococcus* and *β-H. streptococcus* (1 × 10^7^ cfu/ml) were diluted in coating buffer and coated in wells of ELISA plates at 4.0 °C overnight. The ELISA results showed that 14 clones had good specificity with *S. aureus* and no binding reaction with the control bacteria (Fig. 5). Number 15 and 16 (0.01 mol/L PBS was added, not scFvs) were used as the negative controls. Each test was repeated for three times, the value of the blank was removed, and the standard errors were calculated. The scFvs with better binding activity and specificity (S78, S117, S128, S182) were inserted into the pLZ16 vector with human IgG1 Fc.

### Construction and expression of scFv-Fcs

**Fig. 6:**
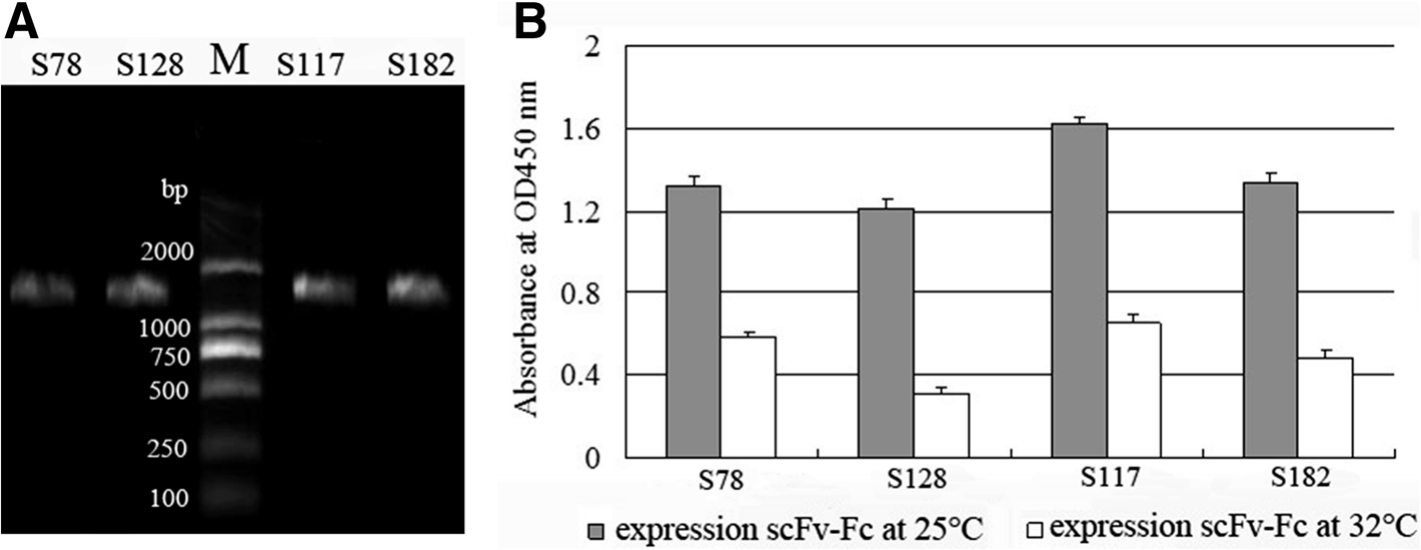
**a** Constructed scFv-Fcs (S78, S128, S117, S182) were amplified and their sizes were ~1.5 kb. Lane M: 2000 bp DNA Marker (TaKaRa, JP). **b** Expression level of scFv-Fcs (S78, S128, S117, S182) expressed at 25 °C and 32 °C, respectively, were tested by enzyme-linked immunosorbent assay

**Fig. 7:**
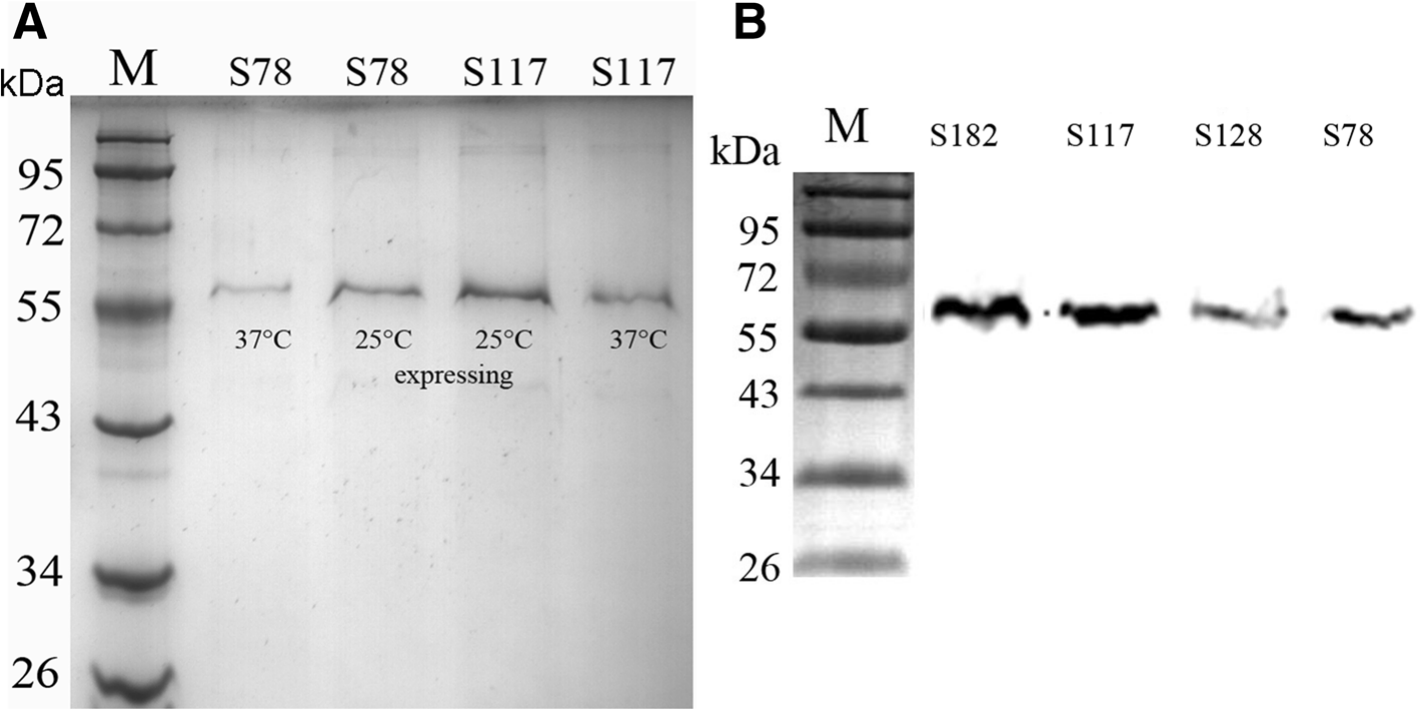
**a** The scFv-Fcs (S78, S117) expressed at 25 °C and 32 °C respectively were purified and run SDS-PAGE. Lane M: prestained protein ladder (Fermentas, USA); **b** The scFv-Fcs (S78, S128, S117, S182) were identified by western blot. Lane M: prestained protein ladder (Fermentas, USA)

To overcome the instability of scFvs, scFv-Fcs were constructed. The Fc region comprises the CH_2_ and CH_3_ domains and the hinge region of the human IgG1. The hinge serves as a flexible spacer between the two parts of the Fc-fusion protein, allowing each part of the molecule to function independently. After transformation, the clones were identified by PCR amplification. The results showed that the bands at 1.5 kb were amplified (Fig. 6a). The sequencing results showed that the scFv-Fcs were correct. Next, the scFv-Fcs were induced to express at 25 °C for 50 h without IPTG or 32 °C for 5.0 h with 1.0 mmol/L IPTG. The expressed scFv-Fcs were coated on the ELISA plates and detected with goat anti-human IgG1 Fc HRP-conjugated antibody. The results showed that OD_450_ of scFv-Fcs expressed at 25 °C was higher than that expressed at 32 °C (Fig. 6b). The positive results of detection with human IgG1 Fc HRP-conjugated antibody showed that the Fc regions were successfully fused into scFv. The expressed scFv-Fc (S78, S117) under the two conditions were purified. From sodium dodecyl sulfate polyacrylamide gel electrophoresis staining (SDS-PAGE) (Fig. 7a), the scFv-Fcs was pure and not degraded after 50 h expression at lower temperatures (25 °C). The lower temperature will degrade the aggregation of expressed protein and stabilize the bioactivity of scFv-Fc. MgCl_2_ was added to the medium to maintain the cell wall. Later, the expressed scFv-Fcs (S78, S117, S128, S182) at 25 °C were detected by western blot with the anti-FLAG HRP-conjugated antibody and a single band of ~67 kDa appeared (Fig. 7b). Based on this, it was concluded that a lower temperature was beneficial to the expression and folding of scFv-Fcs and that the system used is suitable for the production of Fc-fused antibody fragments.

### scFv-Fc functionality

**Fig. 8:**
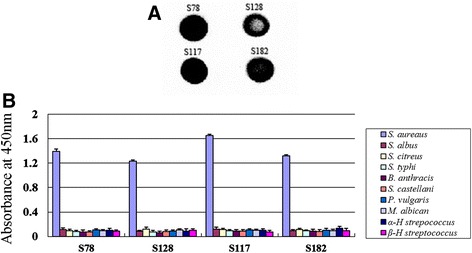
**a** Binding activity of scFv-Fcs (S78, S128, S117, S182) and *S. aureus* were detected by dot blot*.* The immunoreactions was developed by chemiluminscence (**b**). Detection of the specificity of scFv-Fcs (S78, S128, S117, S182) by enzyme-linked immunosorbent assay

**Fig. 9:**
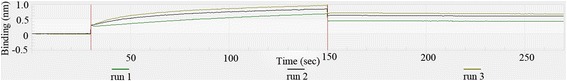
Analysis of the association and dissociation of S117 and *S. aureus*. The S117 was immobilized on AHC biosensor, the different concentration of *S. aureus* were tested. (run1: 1 × 10^7^ cfu/mL, run 2: 2 × 10^7^ cfu/mL, run 3: 4 × 10^7^ cfu/mL)

The dot blot results showed that the scFv-Fcs had good binding activity with the *S. aureus* using the goat anti-human IgG1 Fc HRP-conjugated antibody (Fig. 8a). In addition, the specificity of scFv-Fcs remained and had no cross reaction with *S. aureus, S. albus, S. citreus, S. typhi, B. anthracis, S. castellani, P. vulgaris, M. albicans, α-H. streptococcus* or *β-H streptococcus* (Fig. 8b). The BIAcore (BLITZ) results also showed that the scFv-Fcs (S78, S117, S128, S182) could bind *S. aureus* well (Data not shown). The real-time association and dissociation of S117 and *S. aureus* were showed in Fig. 9, and the equilibrium dissociation constant (K_D_), the rate constants for association (ka) and dissociation (kd) between *S. aureus* and selected scFv-Fcs were listed in Table 1, which were determined by BIAcore (BLITZ).

**Table 1:** The kinetic constants determined by BIAcore (BLITZ) analysis for scFvs

scFv	k_a_ (1/Ms)	k_d_ (1/s)	k_d_/k_a_(K_D_, M)
S78	4.937e4	5.731e-4	1.161e-8
S128	2.123e4	8.231e-4	3.877e-8
S117	5.539e5	4.804e-4	8.673e-9
S182	2.213e4	7.648e-4	3.456e-8

The opsonization of scFv-Fcs was detected. Compared with mouse IgG controls, the plates spread with the mixture containing scFv-Fc (S78, S117) had less clones (Table 2) and it has statistically significant for S78 (*P* < 0.01) and S117 (*P* < 0.001). The results showed the scFv-Fcs (S78, S117) against S. *aureus* could play the role of opsonization, and promoting the function of phagocytosis of macrophage.

**Table 2:** Test of the opsonization of scFv-Fcs

	clones		clones
*S. aureus* + macrophage + S78	(42 ± 5) × 10^4^ **	*S. aureus* + macrophage + IgG	(78 ± 7) × 10^4^
*S. aureus* + macrophage + S128	(62 ± 6) × 10^4^	S.aureus + macrophage	(82 ± 8) × 10^4^
*S. aureus* + macrophage + S117	(26 ± 5)10^4^***		
*S. aureus* + macrophage + S182	(56 ± 6) × 10^4^		

**P <0.01, ***P <0.001

### Discussion

Commercial murine IgG against *S. aureus* has been used in research but no fully human antibody against *S. aureus* has been commercially available for treatment of the infection of *S. aureus*. Our scFv-Fc against *S. aureus* was a fully human antibody, and the affinity of scFv was not evolved in vivo; therefore, after the functional scFv-Fc was selected, the affinity of scFv-Fc will be improved by affinity maturation in vitro and the antigenic determinant of *S. aureus* also will be researched*.* We hope to establish an adjunctive therapy for severe *S. aureus* infections using those antibodies.

scFv comprised only Ig V_H_ and V_L_ connected by a soluble and flexible oligopeptide. scFv use is easy for the design and construction of a phage display antibody library, and phage display technology makes possible the direct isolation of monovalent scFv antibody fragments; however, scFv has disadvantages of being unstable, having a short serum half-life, and lacking avidity because of monovalent binding, all of which could limit its effectiveness. Reducing these disadvantages would be useful in making scFv more successful as an antibody. The scFv-Fc format has certain properties of IgGs, such as bivalency, tag-free detection, and Fc-mediated effector functions. Moreover, scFv-Fc antibodies have been successfully used to neutralize viral, bacterial, and fungal pathogens in vitro and in vivo, which suggests its potential uses in therapy [14–16].

In this study, the scFv against *S aureus* was screened and merged with Fc. The scFv-Fc was expressed with the vector pLZ16 and the better expression condition was incubated at 25 °C for 50 h without IPTG. According to Hiroyoshi (2008) [13], optimum conditions were established for bacterial cultivation and protein expression, utilizing an unusually long cultivation time (>50 h), very low temperature (25 °C), and no IPTG, thereby leading to the production and extracellular secretion of fragment antigen-binding (Fab) proteins in a high-yield using the vector pComb43C9 with lactose promoter.

Based on the report of Hiroyoshi [13], the vector pLZ16 was designed in this study for expressing the soluble scFv-Fc antibody. The results showed that the overall gene expression level under the conditions of 25 °C for 50 h and without IPTG induction was higher than the expression under the conditions of 32 °C for 5.0 h with IPTG. The production yield of the functional scFv-Fc at 25 °C was highly dependent on the following points: (1) freshness of a single colony of the phagemid-harboring *E. coli* cells; (2) temperature controls, including pre-warming of the culture medium in the inoculation steps; and (3) medium exchange to one with no glucose at the induction step for protein expression [13].

Another study reported that auto-induction without IPTG allows efficient screening many clones in parallel for expression and solubility, as cultures need only to be inoculated and grown to saturation, and yields of target protein are typically several-fold higher than that obtained by conventional IPTG induction [17].

The expressed and purified scFv-Fcs have good bioactivity and specificity with *S. aureus*. In subsequent studies, the antigenic determinant mechanisms of action will be researched.

## Conclusions

In this work, a human scFv library with a volume of 1.7 × 10^7^ was constructed. From the library, specific scFvs against *S. aureus* were selected using three rounds of phage display. The scFv-Fcs with stable structure developed and expressed in *E. coli* DH5α, and the suitable expression system induced at 25 °C was beneficial for the correct folding of scFv-Fcs. The purified scFv-Fcs also had the functionality of good binding activity and specificity to *S. aureus.* These antibodies could be used as candidates for the development of future adjunctive therapy for severe *S. aureus* infections.

## Availability of data and materials

Considering the data has not been used for applying patent, the data will not be shared.

## Abbreviations

ADCC: antibody-dependent cellular cytotoxicity
ELISA: enzyme-linked immunosorbent assay
Fc: fragment crystallizable region
FCS: fetal calf serum
Ig: immunoglobulin
IPTG: isopropyl β-D-1-thiogalactopyranoside
HAMA: human anti-mouse antibody
MRSA: meticillin resistant *Staphylococcus aureus*
PBMC: peripheral blood mononuclear cell
PBS: phosphate-buffered saline
PCR: polymerase chain reaction
PEG: polyethylene glycol
scFv: single-chain variable fragment
V_H_: variable regions of heavy-chain
V_L_: variable regions of light-chain
